# Molecular Imaging of Collagen Turnover in Myocardial Infarction

**DOI:** 10.2967/jnumed.125.271721

**Published:** 2026-06

**Authors:** Afarin Neishabouri, Mean Ghim, Onur Varli, Azmi A. Ahmad, Gunjan Kukreja, Zhengxing Zhang, Jie Li, Jakub Toczek, Mani Salarian, Kiran Gona, Keshvad Hedayatyanfard, Jiasheng Zhang, Daniel Ein Alshaeba, Fadi G. Akar, Chi Liu, Henry Huang, S. Michael Yu, Mehran M. Sadeghi

**Affiliations:** 1 Yale Cardiovascular Research Center, Section of Cardiovascular Medicine, Department of Internal Medicine, Yale School of Medicine, New Haven, Connecticut;; 2 Veterans Affairs Connecticut Healthcare System, West Haven, Connecticut;; 3 Department of Radiology and Biomedical Imaging, Yale University, New Haven, Connecticut; and; 4 Department of Biomedical Engineering and Department of Molecular Pharmaceutics, University of Utah, Salt Lake City, Utah

**Keywords:** fibrosis, cardiomyopathy, myocardial infarction, molecular imaging, SPECT/CT

## Abstract

Cardiac fibrosis is a key contributor to cardiomyopathy after myocardial infarction (MI). Existing imaging techniques can detect established fibrotic changes; however, they lack sensitivity for ongoing collagen turnover—a dynamic process involving the denaturation of collagen triple helix. Molecular imaging of this process could enhance risk assessment and aid in the development of antifibrotic treatments. This study aimed to evaluate ^99m^Tc-(HE)_3_-(GPO)_9_, a radiotracer designed to target denatured collagen, as a biomarker of collagen turnover after MI. This tracer features a polyhistidine–glutamic acid [(HE)_3_] N-terminal sequence for site-specific radiolabeling linked to a C-terminal–targeting moiety consisting of 9 glycine–proline–hydroxyproline repeats [(GPO)_9_] via a flexible 3-glycine linker. **Methods:** MI was induced in mice by ligation of the left anterior descending artery; animals who underwent sham surgery served as controls. At 2 wk after MI, animals underwent myocardial perfusion imaging or contrast-enhanced CT to detect the infarct zone, followed by SPECT/CT imaging with ^99m^Tc-(HE)_3_-(GPO)_9_ or a control tracer with scrambled peptide. Tracer uptake was quantified in vivo and ex vivo with γ-counting and autoradiography. Different aspects of fibrosis were examined using tissue analysis, along with autoradiography with a matrix metalloproteinase–targeted radiotracer, ^99m^Tc-RYM1, at 3 d, 1 wk, and 2 wk after MI. Tracer binding was also assessed in human cardiac tissue using ex vivo autoradiography. **Results:** ^99m^Tc-(HE)_3_-(GPO)_9_ SPECT/CT revealed significantly higher tracer uptake in the infarct zone of MI mice compared with the remote zone and sham controls (*P* < 0.0001 for both). Tracer uptake was confirmed by autoradiography, which showed a strong correlation between SPECT and autoradiography (*ρ* = 0.81, *P* < 0.05). The control tracer exhibited minimal cardiac uptake, demonstrating the specificity of the ^99m^Tc-(HE)_3_-(GPO)_9_ signal. Denatured collagen staining and ^99m^Tc-RYM1 autoradiography showed patterns similar to that shown in ex vivo ^99m^Tc-(HE)_3_-(GPO)_9_ autoradiography, whereas the ratio of denatured collagen to procollagen in the infarct zone significantly increased from day 3 to 2 wk after MI. Finally, ^99m^Tc-(HE)_3_-(GPO)_9_ demonstrated binding to human fibrotic (but not normal) cardiac tissue. **Conclusion:** ^99m^Tc-(HE)_3_-(GPO)_9_ enabled noninvasive detection of denatured collagen after MI as a marker of collagen remodeling in vivo. In combination with other fibrosis imaging tracers, ^99m^Tc-(HE)_3_-(GPO)_9_ may provide a comprehensive molecular fingerprint of cardiac fibrosis, advancing personalized management of cardiomyopathy.

## Visual Abstract

**Figure:**
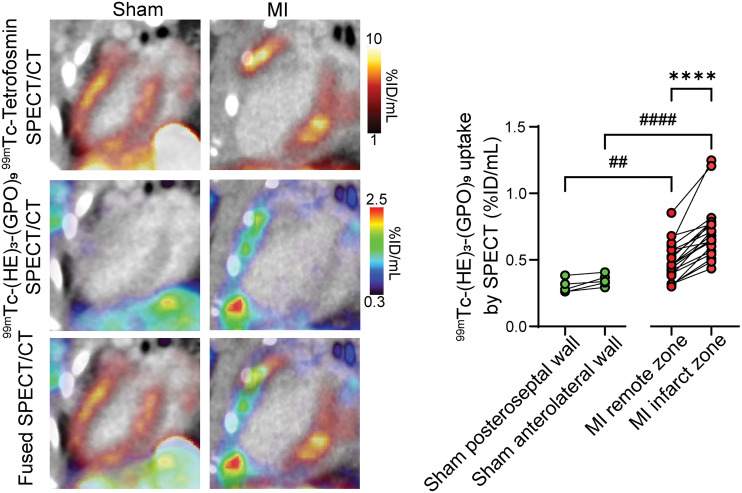

Dysregulated tissue repair in response to injury results in fibrosis, a chronic, progressive process characterized by excessive deposition of extracellular matrix proteins, including collagen (1). Myocardial fibrosis is associated with a wide range of cardiac pathologies, including ischemic and nonischemic cardiomyopathies (2). Ischemic events, such as myocardial infarction (MI), result in replacement fibrosis, the recruitment of inflammatory cells, and the activation of matrix-producing myofibroblasts. Although the resulting collagen-based scar tissue protects the infarcted ventricle from mechanical complications, such as cardiac rupture, it may contribute to ventricular remodeling, especially when associated with interstitial fibrosis in the remote zone, and lead to systolic and diastolic dysfunction and potentially heart failure (3). Detection of the processes involved in fibrosis and tracking its progression and regression can accelerate the development and evaluation of new antifibrotic therapies and help stratify patients for targeted personalized antifibrotic treatments (4).

Noninvasive imaging can detect cardiac fibrosis and its consequences, such as changes in ventricular function and geometry, primarily using echocardiography and MRI (5,6). Myocardial perfusion imaging (MPI), using PET or SPECT, detects the perfusion deficit associated with scarring (7). Newly introduced molecular imaging approaches can evaluate the established fibrosis and fibrosis-associated processes by targeting integrins, collagen, fibroblast activation protein (FAP), or proteolytic enzymes (8–11). However, none of these approaches directly detects the changes in collagen structure that mediate collagen turnover.

Collagen has a unique triple-helical structure that unfolds after enzymatic cleavage (e.g., by matrix metalloproteinase [MMP]) or mechanical damage. Collagen hybridizing peptides (CHPs) are a family of synthetic peptides with repetitive collagen-mimetic sequences, such as glycine–proline–hydroxyproline [(GPO)]. CHPs can hybridize to 1 or 2 unfolded collagen chains to form a stable triple helix, enabling detection of collagen remodeling during fibrosis (12–14). We recently introduced a CHP-based radiotracer, ^99m^Tc-(HE)_3_-(GPO)_9_, to detect denatured collagen by SPECT/CT imaging. This radiotracer features a polyhistidine–glutamic acid [(HE)_3_] N-terminal sequence for site-specific radiolabeling linked to a C-terminal–targeting moiety consisting of 9 GPO repeats [(GPO)_9_] via a flexible 3-glycine linker (15). In the current study, we investigated the performance of ^99m^Tc-(HE)_3_-(GPO)_9_ for molecular imaging of collagen turnover in murine MI.

## MATERIALS AND METHODS

Detailed materials and methods are provided in the supplemental materials, available at http://jnm.snmjournals.org (16,17).

### Radiotracers

The (HE)_3_-(GPO)_9_ precursor was synthesized and radiolabeled to generate ^99m^Tc-(HE)_3_-(GPO)_9_ as previously described (15). A control tracer, ^99m^Tc-(HE)_3_-(GPO)_SCR_, in which (GPO)_9_ was replaced with a scrambled sequence (PGOGPGPOPOGOGOPPGOOPGGOOPPG), served as a negative control agent.

### Animals

Animal experiments were performed in accordance with protocols approved by the Institutional Animal Use and Care Committees of Yale University (10455) and the Veterans Affairs Connecticut Healthcare System (MS0003). To induce MI, 10- to 16-wk-old C57BL/6J mice (*n* = 39, Supplemental Fig. 1) of both sexes underwent left anterior descending artery ligation 2 mm distal to the left auricle, as described, with some modifications (18). Sham-operated or unoperated animals (*n* = 8, Supplemental Fig. 1) served as the control group.

### SPECT/CT Imaging

A subset of animals underwent ^99m^Tc-tetrofosmin MPI 2 wk after MI or a sham operation (Supplemental Fig. 1). Animals were anesthetized with 1%–3% isoflurane, retroorbitally injected with 26.3 ± 4.1 MBq of ^99m^Tc-tetrofosmin, and imaged 20 min after radiotracer injection. SPECT image acquisition was performed on a small-animal SPECT/CT scanner (U-SPECT4CT; MILabs) for 15 min, followed by a full-body CT scan, as previously described (15). Animals were allowed to rest for 2 or 3 d after ^99m^Tc-tetrofosmin imaging to allow the radioactivity to clear before imaging with ^99m^Tc-(HE)_3_-(GPO)_9_. ^99m^Tc-(HE)_3_-(GPO)_9_ SPECT/CT imaging was performed 1 h after retroorbital injection of 29.2 ± 7.8 MBq of heated (to 80 °C for 10 min) and cooled (on ice for 30 s) ^99m^Tc-(HE)_3_-(GPO)_9_ and 30 µL plus 1 µL per gram of body weight of Exitron Nano-12000 (Viscover). The time point of 1 h point was selected on the basis of a prior study of ^99m^Tc-(HE)_3_-(GPO)_9_, which showed a residual blood activity of less than 1 %ID/mL at 60 min and a small decline in blood levels between 60 and 120 min (15). List-mode images were obtained for 45 min. Instead of undergoing tetrofosmin MPI, a subset of animals was injected retroorbitally with 50 µL plus 1 µL per gram of body weight of eXIA 160XL (Binitio Biomedical Inc.), a CT contrast agent that is taken up by viable myocardium (19), and imaged 210–270 min later by CT to identify the infarct zone. To address tracer uptake specificity, a subset of animals underwent ^99m^Tc-(HE)_3_-(GPO)_9_ SPECT (23.3 ± 6.7 MBq) imaging, followed by ^99m^Tc-(HE)_3_-(GPO)_SCR_ SPECT (26.3 ± 6.3 MBq) within 2 or 3 d. Each SPECT study was coupled with either eXIA 160XL enhanced CT or Exitron Nano-12000 enhanced CT.

MILabs software version 12 was used to reconstruct contrast-enhanced CT (ceCT) images at an isotropic voxel size of 0.1 mm using a filtered backprojection algorithm. Emission data were reconstructed using a similarity-regulated ordered-subsets expectation maximization algorithm with a 0.4-mm voxel size, 16 subsets, 9 iterations, and a 0.45-mm full-width-at-half-maximum gaussian filter. The SPECT data were then registered to the ceCT images, and the images were quantified using 3D Slicer software (version 5.2.2) and visualized with AMIDE software (version 1.0.6). Segmentations of infarct and remote zones or their corresponding left ventricular walls in sham-operated animals were performed on the basis of tetrofosmin MPI or eXIA 160XL ceCT images. We identified the infarct and remote zones using tetrofosmin MPI or Exia 160 XL ceCT images, generating a volume of interest for each zone. This volume of interest was then applied to ^99m^Tc-(HE)_3_-(GPO)_9_ images, excluding any location where the uptake in the surgical wound or the liver could not be differentiated from the cardiac signal.

### Biodistribution and Autoradiography

Blood samples were obtained after SPECT/CT imaging and 2 h after tracer injection, and the animals were euthanized. Tissue samples were collected from the heart apex and several other organs, and their radioactivity was quantified, using a γ-well counter (Wizard2; PerkinElmer) as the percentage of injected dose per gram of body weight, along with the blood’s activity, measured as percentage of injected dose per milliliter. The heart was cut into 4 segments along its long axis, and the apical part was used for γ-counting. The remaining heart segments were embedded in optimal cutting temperature compound, stored on dry ice, and sectioned on the same day. For quantitative autoradiography, 10-µm-thick sections and references of known activity were exposed to a phosphor screen (MultiSensitive Phosphor Screen; PerkinElmer) and subsequently scanned with a phosphor imager (Typhoon Trio; GE HealthCare Life Sciences). The injected dose was calibrated to a set time to account for decay from the time of injection to the time of exposure. The tracer signals in the remote and infarct zones (and corresponding walls of sham-operated animals) were measured on calibrated images using Fiji/ImageJ software, and the results were expressed as percentage of injected dose per square centimeter. Other tissue sections—obtained from controls, 3d after MI, 1 wk after MI, or 2 wk after MI following the initial decay of radioactivity—were used for ^99m^Tc-(HE)_3_-(GPO)_9_ or ^99m^Tc-RYM1 autoradiography. Tissue sections of 7-µm thickness were fixed in 10% neutral buffered formalin and blocked with 5% bovine serum albumin. Sections were then incubated with 0.57 ± 0.04 MBq (in 600–800 µL) of ^99m^Tc-(HE)_3_-(GPO)_9_ at 37 °C or ^99m^Tc-RYM1 at room temperature for 60 min, followed by 3 washes in phosphate-buffered saline for 5 min each. The slides and known radioactivity standards were exposed to a phosphor plate overnight and scanned with a phosphor imager. For human tissue autoradiography, the slides were deparaffinized and rehydrated in xylene for 10 min twice, then 100% ethanol for 10 min twice, followed by 75% ethanol for 5 min and 50% ethanol for 5 min. Finally, they were rinsed in phosphate-buffered saline. Trichrome images were analyzed in ImageJ to generate a binary mask of blue collagen, which was then overlaid onto autoradiography.

## RESULTS

### Radiochemistry

^99m^Tc-(HE)_3_-(GPO)_9_ and its nonbinding tracer (control), ^99m^Tc-(HE)_3_-(GPO)_SCR_, were radiolabeled with ^99m^Tc-tricarbonyl and purified to a specific activity of 83.9 ± 35.5 GBq/µmol and 88.1 ± 27.2 GBq/µmol, respectively, and their labeling efficiency and purity were confirmed by radio–high-performance liquid chromatography (Supplemental Fig. 2).

### ^99m^Tc-(HE)_3_-(GPO)_9_ SPECT/CT in MI

**FIGURE 1.**
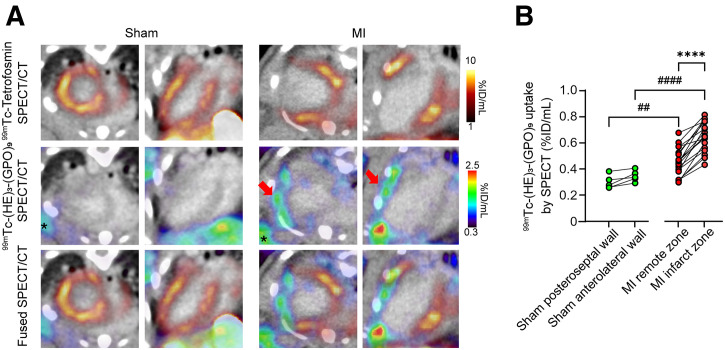
^99m^Tc-(HE)_3_-(GPO)_9_ SPECT/CT in MI. (A) Illustrative examples of in vivo SPECT/Exitron Nano-12000–enhanced CT images of sham-operated and MI-induced mice at 2 wk after surgery. Arrows in middle column indicate ^99m^Tc-(HE)_3_-(GPO)_9_ uptake in infarct zone of MI-induced mice, identified by ^99m^Tc-tetrofosmin MPI (top row). Single asterisks mark ^99m^Tc-(HE)_3_-(GPO)_9_ uptake at surgical site in both sham and MI-induced mice. (B) Quantification of ^99m^Tc-(HE)_3_-(GPO)_9_ SPECT signal in infarct and remote zones of MI-induced mice and corresponding walls of sham-operated mice. ^##^*P* < 0.01 (Mann–Whitney *U* test); ^####^*P* < 0.0001 (Mann–Whitney *U* test); ****P < 0.0001 (Wilcoxon signed-rank test).

Echocardiography at 2 wk after left anterior descending artery ligation showed the presence of regional wall motion abnormalities and a significant reduction in left ventricular ejection fraction, when compared with sham-operated animals (*P* < 0.01) (Supplemental Fig. 3). ^99m^Tc-tetrofosmin MPI or eXIA 160XL ceCT imaging at 2 wk after surgery confirmed the presence of an anterolateral infarct area in MI animals, whereas, as expected, sham-operated animals did not show any perfusion defect (Fig. 1A). MPI was followed by ^99m^Tc-(HE)_3_-(GPO)_9_ small-animal SPECT/CT imaging 2–3 d apart, starting at 60 min after tracer injection. ^99m^Tc-(HE)_3_-(GPO)_9_ SPECT/CT images showed markedly higher uptake of the tracer in the infarct zone in MI-induced animals compared with their remote zone and with sham-operated mice (Fig. 1A). This was confirmed by quantifying the left ventricular wall ^99m^Tc-(HE)_3_-(GPO)_9_ signal on SPECT/CT images (0.65 %ID/mL; range, 0.56–0.76 %ID/mL) for the infarct zone versus 0.46 %ID/mL (range, 0.37–0.54 %ID/mL) for the remote zone in the MI group (*n* = 18, *P* < 0.0001) and 0.34 %ID/mL (range, 0.31–0.39 %ID/mL, *n* = 5) in the corresponding anterolateral wall of sham-operated animals (*P* < 0.0001). There was no difference in tracer uptake between the anterolateral and posteroseptal walls of sham-operated animals (Fig. 1B).

### ^99m^Tc-(HE)_3_-(GPO)_9_ Biodistribution

Ex vivo evaluation by γ-well counting of tissues collected from animals euthanized 2 h after tracer administration showed significantly higher tracer uptake in the apex of MI mice compared with the sham group (1.31 %ID/g vs. 0.46 %ID/g, *P* < 0.01), with no difference in residual blood radioactivity. Furthermore, tracer uptake in the lungs, liver, spleen, kidneys, white adipose tissue, and muscle did not significantly differ between the sham and MI groups (Supplemental Figs. 4A–4E). Importantly, there was a significant correlation between SPECT-based quantification of tracer uptake in the apex and ex vivo, γ-well counting–based quantification of tracer uptake in the same animals (Spearman ρ = 0.71, *P* < 0.05) (Supplemental Fig. 4F), indicating the accuracy of in vivo tracer uptake quantification.

### ^99m^Tc-(HE)_3_-(GPO)_9_ Autoradiography

**FIGURE 2.**
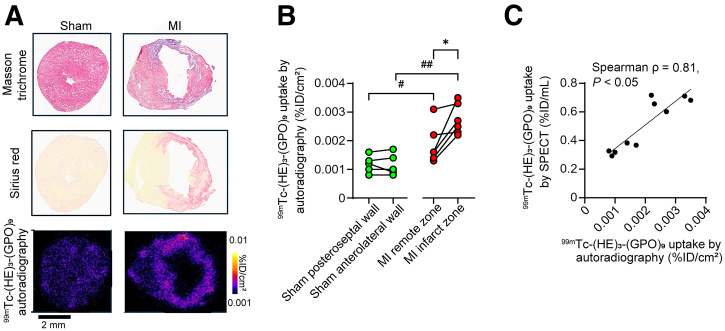
^99m^Tc-(HE)_3_-(GPO)_9_ autoradiography in MI. (A) Illustrative examples of Masson trichrome staining, Sirius red staining, and ^99m^Tc-(HE)_3_-(GPO)_9_ autoradiography (after in vivo tracer administration) of sham-operated and MI-induced mice 2 wk after surgery. (B) Quantification of ^99m^Tc-(HE)_3_-(GPO)_9_ uptake by autoradiography in infarct and remote zones of MI-induced mice and corresponding walls of sham-operated mice. (C) Correlation between ^99m^Tc-(HE)_3_-(GPO)_9_ signals on SPECT/CT and autoradiography in infarct zone and anterolateral wall of sham-operated mice. ^#^*P* < 0.05 (Mann–Whitney *U* test); *P < 0.05 (Wilcoxon signed-rank test); ^##^*P* < 0.01 (Mann–Whitney *U* test).

Evaluation of in vivo tracer uptake by autoradiography showed significantly higher ^99m^Tc-(HE)_3_-(GPO)_9_ uptake in the infarct zone, detected by Masson trichrome and Sirius red staining (0.0026 %ID/cm^2^; range, 0.0023–0.0035 %ID/cm^2^), compared with the remote zone of MI-induced mice (0.0016 %ID/cm^2^; range, 0.0014–0.0024 %ID/cm^2^; *n* = 6; *P* < 0.05) and with the corresponding wall in the sham group (0.0010 %ID/cm^2^; range, 0.0009–0.0017 %ID/cm^2^; *n* = 5; *P* < 0.01) (Figs. 2A and 2B). Notably, there was a significant correlation between in vivo SPECT-based quantification of tracer uptake in the infarct zone (or the corresponding anterolateral left ventricular wall in the sham-operated animals) and ex vivo autoradiography-based quantification of tracer uptake in the same animals, confirming the accuracy of in vivo tracer uptake measurements (Spearman ρ = 0.81, *P* < 0.05 (Fig. 2C).

### Specificity of Tracer Uptake

**FIGURE 3.**
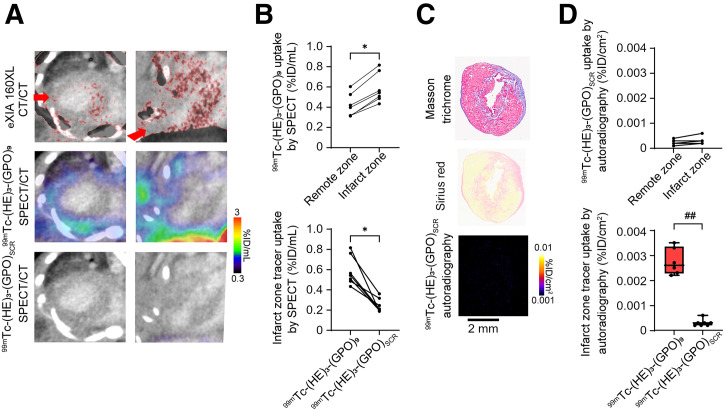
^99m^Tc-(HE)_3_-(GPO)_9_ specificity. (A) Illustrative examples of in vivo ceCT using eXIA 160XL fused with Exitron Nano-12000–enhanced CT (top row), along with SPECT/Exitron Nano-12000–enhanced CT of ^99m^Tc-(HE)_3_-(GPO)_9_ (middle row) and ^99m^Tc-(HE)_3_-(GPO)_SCR_ (bottom row) in MI-induced mice at 2 wk after surgery. Arrows point to scarred area. (B) Quantification of ^99m^Tc-(HE)_3_-(GPO)_9_ SPECT signal in infarct zone of MI-induced mice compared with ^99m^Tc-(HE)_3_-(GPO)_9_ signal in remote zone (top graph) and ^99m^Tc-(HE)_3_-(GPO)_SCR_ signal in infarct zone (bottom graph) of same mice. (C) Illustrative examples of Masson trichrome staining, Sirius red staining, and ^99m^Tc-(HE)_3_-(GPO)_SCR_ autoradiography (after in vivo tracer administration) of MI-induced mice 2 wk after surgery. (D) Quantification of ^99m^Tc-(HE)_3_-(GPO)_SCR_ uptake by autoradiography in infarct zone of MI-induced mice compared with ^9m^Tc-(HE)_3_-(GPO)_SCR_ uptake in remote zone (top graph) and ^99m^Tc-(HE)_3_-(GPO)_9_ uptake in infarct zone (bottom graph) of same animals. *P < 0.05 (Wilcoxon signed-rank test); ^##^*P* < 0.01 (Mann–Whitney *U* test).

To investigate the specificity of ^99m^Tc-(HE)_3_-(GPO)_9_ signal in MI, a group of mice in whom MI was induced (*n* = 7) underwent repeat small-animal SPECT/CT-CT imaging with ^99m^Tc-(HE)_3_-(GPO)_9_ and ^99m^Tc-(HE)_3_-(GPO)_SCR_ within 2 or 3 d after tracer administration. CT imaging with eXIA 160XL, a CT contrast agent that is taken up by viable myocardium (19), identified the infarct zone in these mice. The control tracer and ^99m^Tc-(HE)_3_-(GPO)_9_ showed different patterns of uptake throughout the heart (Fig. 3A). Quantification of the infarct zone SPECT signal showed significantly higher uptake of ^99m^Tc-(HE)_3_-(GPO)_9_ (mean, 0.54 %ID/mL; range, 0.49–0.56 %ID/mL) versus ^99m^Tc-(HE)_3_-(GPO)_SCR_ (mean, 0.21 %ID/mL; range, 0.20–0.32 %ID/mL; *P* < 0.05) (Fig. 3B), establishing the specificity of the ^99m^Tc-(HE)_3_-(GPO)_9_ signal in MI. Evaluation of tracer uptake by autoradiography showed that, while there was no difference in ^99m^Tc-(HE)_3_-(GPO)_SCR_ uptake in vivo between the infarct and remote zones of the same hearts, the infarct zone ^99m^Tc-(HE)_3_-(GPO)_SCR_ signal was significantly lower than the ^99m^Tc-(HE)_3_-(GPO)_9_ signal (*P* < 0.01; Figs. 3C and 3D).

### Correlates of Cardiac ^99m^Tc-(HE)_3_-(GPO)_9_ Uptake

**FIGURE 4.**
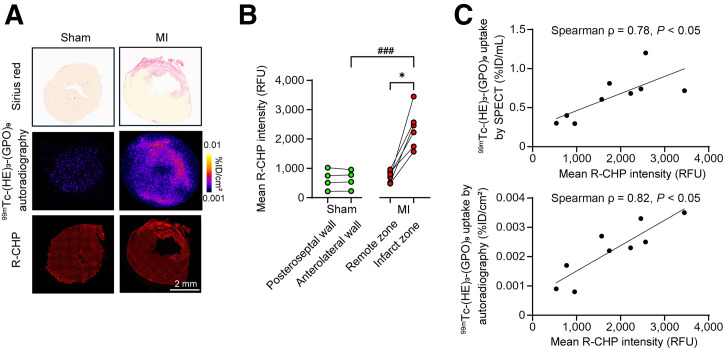
(A) Illustrative examples of Sirius red staining, ^99m^Tc-(HE)_3_-(GPO)_9_ autoradiography (after in vivo tracer administration), and denatured collagen staining of sham-operated and MI-induced mice at 2 wk after surgery using R-CHP. (B) Quantification of R-CHP staining in MI-induced and sham-operated animals. (C) Correlation between ^99m^Tc-(HE)_3_-(GPO)_9_ uptake by SPECT/CT (top) or autoradiography (bottom), and R-CHP staining intensity in infarct zone. RFU: relative fluorescence unit. *P < 0.05 (Wilcoxon signed-rank test); ^###^*P* < 0.001 (Mann–Whitney *U* test).

Evaluation of denatured collagen using a fluorescent CHP (R-CHP), when compared with Sirius red and Masson trichrome staining (both of which detect collagen), showed that the quantity of denatured collagen was significantly higher in the infarct zone (*P* < 0.05 compared with the remote zone or the corresponding wall in sham-operated animals at the 2-wk time point (Figs. 4A and 4B). Notably, at 2 wk after MI, the amount of denatured collagen quantified by R-CHP staining in the infarct zone and in the corresponding anterolateral left ventricular wall of sham-operated animals was significantly correlated with ^99m^Tc-(HE)_3_-(GPO)_9_ uptake by SPECT/CT (Spearman ρ = 0.78, *P* < 0.05) and autoradiography (Spearman ρ = 0.82, *P* < 0.05) (Fig. 4C).

### Denatured Collagen and Other Targets for Imaging Remodeling

**FIGURE 5.**
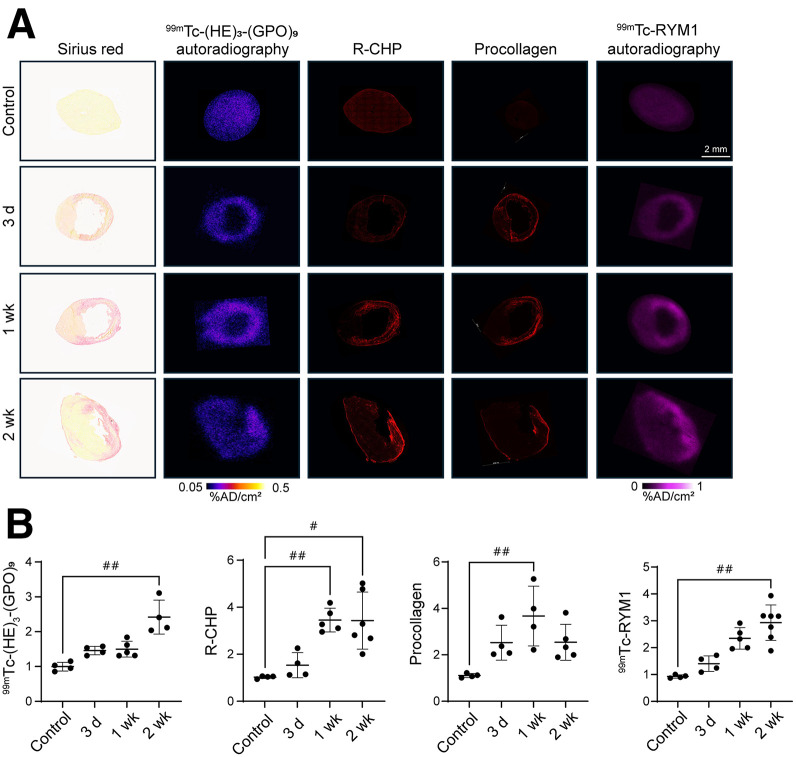
Fibrosis-related imaging targets over time. Illustrative examples (A) and infarct–to–remote area signal intensity ratios (B) of ex vivo ^99m^Tc-(HE)_3_-(GPO)_9_ autoradiography, R-CHP staining, procollagen staining, and ex vivo ^99m^Tc-RYM1 autoradiography in adjacent sections of hearts collected from controls and from MI-induced mice at 3 d, 1 wk, and 2 wk after MI. Sirius red images are shown as reference in panel A. AD = applied dose. ^#^*P* < 0.05 (Kruskal–Wallis test); ^##^*P* < 0.01 (Kruskal–Wallis test).

Next, we evaluated the presence of denatured collagen by ex vivo ^99m^Tc-(HE)_3_-(GPO)_9_ autoradiography and R-CHP staining at 3 d, 1 wk, and 2 wk after MI and compared it with controls. Both approaches showed a higher signal in the infarct zone, with the ratio of infarct to remote zone increasing over time (Fig. 5). These changes paralleled the changes in MMP activation detected by ex vivo ^99m^Tc-RYM1 autoradiography (20). Interestingly, whereas procollagen expression in the infarct zone was readily detectable 3 d after MI, the ratio of denatured collagen (reflecting collagen turnover) to procollagen expression significantly increased from 3 d to 2 wk after MI (Supplemental Fig. 5), highlighting the distinct biology of these markers of fibrosis.

### Human Tissue Analysis

**FIGURE 6.**
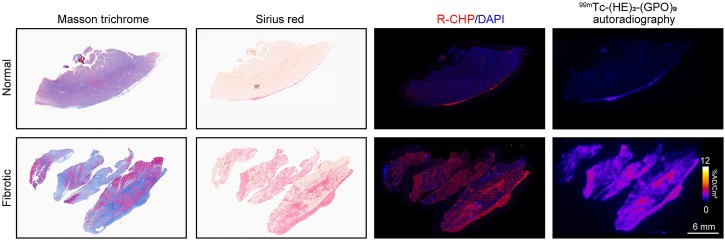
^99m^Tc-(HE)_3_-(GPO)_9_ binding to human myocardial tissue. Illustrative examples of Masson trichrome staining of fibrosis, Sirius red staining of collagen, denatured collagen staining using R-CHP, and ^99m^Tc-(HE)_3_-(GPO)_9_ autoradiography in normal and fibrotic myocardium. DAPI = 4′,6-diamidino-2-phenylindole.

As a prelude to future human studies, we evaluated the binding of ^99m^Tc-(HE)_3_-(GPO)_9_ to normal and fibrotic human myocardial tissues from patients undergoing myocardial biopsy. The presence of fibrosis was confirmed by Masson trichrome and Sirius red staining. The normal myocardium showed little denatured collagen, as detected by R-CHP staining. In contrast, R-CHP staining of the fibrotic biopsies showed a heterogeneous pattern, with denatured collagen localized to fibrotic regions identified by Masson trichrome and Sirius red staining. Similarly, whereas there was minimal ^99m^Tc-(HE)_3_-(GPO)_9_ binding to the normal myocardium, the fibrotic biopsies showed a heterogeneous pattern of ^99m^Tc-(HE)_3_-(GPO)_9_ binding localized to the fibrotic areas (Fig. 6).

## DISCUSSION

Using a recently developed collagen hybridizing tracer, ^99m^Tc-(HE)_3_-(GPO)_9_, we demonstrated the feasibility of in vivo imaging of denatured collagen as a marker of collagen turnover after MI. SPECT/CT images showed higher tracer uptake in the infarct zone compared with the remote zone in mice with MI and in the corresponding left ventricular region in sham-operated animals. The accuracy of in vivo signal quantification was established by comparison with ex vivo autoradiography and γ-counting, and the specificity of tracer uptake was confirmed by comparison with the uptake of a control homolog tracer. Evaluation of denatured collagen by histology showed a strong correlation with in vivo tracer uptake. Notably, various tissue markers of fibrosis exhibited distinct temporal and spatial patterns in MI, suggesting their complementarity as molecular imaging targets. Finally, we demonstrated that the tracer binds effectively to denatured human collagen in fibrotic myocardial biopsies.

Fibrosis is a key characteristic of maladaptive ventricular remodeling (cardiomyopathy), that underlies the development of most heart failure cases. It is often triggered by cardiac injury and mediated by activated cardiac fibroblasts that deposit fibrotic tissue within the myocardium (21). Classically, cardiac fibrosis is categorized as either reactive interstitial fibrosis or replacement fibrosis (21). Replacement fibrosis is the initial response to MI, where there is extensive cardiomyocyte death followed by a repair process that prevents cardiac rupture. This repair process follows the immediate phase of inflammatory cell recruitment and eventually may be associated with remote-zone reactive interstitial fibrosis and maladaptive ventricular remodeling (22). Given the role of fibrosis in cardiac remodeling and arrhythmias, there is considerable interest in new therapies that can prevent or reverse cardiac fibrosis (21). The success of these efforts is dependent on access to noninvasive, quantitative tools to track the development and resolution of cardiac fibrosis and ventricular remodeling.

Typically, the diagnosis of cardiac fibrosis is made on the basis of gadolinium-enhanced MRI findings. Alternative approaches include MPI or other nuclear imaging techniques and integrated backscatter by ultrasound (23). However, these techniques provide a snapshot of the cardiac structure at a given point, without providing any information on disease activity, which is arguably the primary target of therapeutic interventions aimed at preventing progression and promoting regression of fibrosis. Additionally, there is a lag before the effects of therapeutic interventions are reflected in cardiac structure and function and thus become detectable by MRI or ultrasound. Accordingly, novel tools are needed to detect collagen turnover during fibrosis, select patients for emerging therapies, track the effects of therapeutic interventions to guide their timing, and potentially improve prognostication.

To address this unmet need, several new tracers for molecular imaging of fibrosis-related processes have been introduced in recent years. These agents target integrins, fibroblasts, or matrix components, such as mature collagen (24). However, none of these provides adequate information on collagen denaturation and degradation, a key component of extracellular matrix remodeling and a target of therapeutic interventions in fibrosis. Fibroblast activation can be imaged using FAP inhibitor tracers (25). By targeting fibroblast activation, FAP-targeted imaging detects an early stage of fibrosis, with the signal declining after the acute phase of MI (26–28). The extent to which fibroblast activation and the FAP signal are affected by interventions that stabilize or reverse fibrosis has not been determined. Emerging techniques for imaging the activation of proteases involved in tissue remodeling, such as MMPs (18,20,29,30), may partly address these issues; however, at best, they would provide an indirect measure of the downstream effect (i.e., matrix remodeling). ^99m^Tc-(HE)_3_-(GPO)_9_ imaging addresses this unmet need by targeting denatured collagen. The imaging of denatured collagen with MMP and FAP imaging may provide complementary information regarding post-MI fibrosis.

The hallmark of collagen structure is the triple helix, a right-handed helix of 3 α-chains. α-chains are formed by repetitive glycine–X–Y (where X and Y are frequently proline and hydroxyproline) tripeptide motifs, which self-assemble to form collagen fibers (31). The α-chains are highly organized in mature collagen fibers, and single-strand α-chains are present in the extracellular space only when collagen is undergoing degradation, such as by MMPs and cathepsins (31). CHPs, such as the (GPO)_9_ moiety in ^99m^Tc-(HE)_3_-(GPO)_9_, have high triple helical propensity and can bind to 1 or 2 unfolded collagen chains through triple helix formation, allowing specific detection of collagens undergoing degradation (12).

Little information is available regarding MI-induced spatial and temporal changes in denatured collagen (32). Our evaluation of denatured collagen after MI using R-CHP showed an early increase in denatured collagen in the infarct zone compared with the remote zone, which increased from 3 d to 2 wk. Later changes in denatured collagen in both the infarct and remote zones, and their relation to ventricular remodeling, could be a subject of future studies. Notably, there were considerable differences in denatured collagen content between different mice, especially at later time points, which potentially reflects the variability of response to left anterior descending artery ligation in this murine model and highlights the value of longitudinal studies to establish the relationship between denatured collagen and left ventricular structure and function.

As a key step toward such studies, we demonstrated the feasibility of in vivo imaging of denatured collagen as a marker of post-MI collagen turnover. SPECT/CT images showed higher tracer uptake in the infarct zone compared with the remote zone in mice with MI and in the corresponding left ventricular region in sham-operated animals. The specificity of tracer uptake was confirmed by comparing it with the uptake of a control homolog tracer. The tracer uptake in the surgical wound and the liver made quantification of cardiac tracer uptake somewhat challenging. Based on the images obtained with the control tracer, the targeted tracer uptake in the wound was specific, reflecting collagen remodeling during wound healing. Similarly, a large component of liver uptake appeared to be specific, potentially reflecting growth-related remodeling in these young mice. Despite these challenges, the accuracy of in vivo signal quantification was established through comparison with ex vivo autoradiography and γ-counting. Furthermore, histologic evaluation of denatured collagen showed a strong correlation with in vivo tracer uptake. Together, these findings established the feasibility and validity of ^99m^Tc-(HE)_3_-(GPO)_9_ SPECT/CT for detecting denatured collagen after MI. The binding of ^99m^Tc-(HE)_3_-(GPO)_9_ to human denatured collagen in fibrotic myocardial biopsies, although not unexpected given the similarity of collagen structure across species, supports the potential of this family of tracers for imaging in humans.

The similarity between the spatial and temporal patterns of denatured collagen and MMP activation, as revealed by ^99m^Tc-RYM1 autoradiography, highlights the role of MMPs in collagen remodeling. On the basis of these results, one can speculate that the imaging of MMPs and denatured collagen will provide similar information about post-MI ventricular remodeling. Whether this similarity persists in an antifibrotic therapy setting remains to be determined. The distinct patterns of procollagen expression and denatured collagen, on the other hand, reflect their distinct biology in the development and resolution of fibrosis. The significant increase in the ratio of denatured collagen to procollagen expression from 3 d to 2 wk after MI indicates a shift from collagen production to collagen remodeling within this period.

This study had several limitations. The long-term changes in collagen denaturation, the functional significance of the denatured collagen signal after MI, and the effect of antifibrotic therapies on the CHP signal should be addressed in future studies. Although validation in a murine model facilitated future mechanistic studies of collagen denaturation using genetically modified mice, the small size of the left ventricle limited our ability to evaluate regional changes in imaging targets in more detail. In this regard, the detection and quantification of cardiac signal would be facilitated by the development of tracers with reduced nonspecific liver uptake.

## CONCLUSION

^99m^Tc-(HE)_3_-(GPO)_9_ enabled noninvasive detection of denatured collagen as a marker of collagen remodeling in vivo. In combination with other fibrosis imaging tracers, ^99m^Tc-(HE)_3_-(GPO)_9_ may provide a comprehensive molecular fingerprint of cardiac fibrosis, advancing personalized management of cardiomyopathy.

## DISCLOSURE

Mehran Sadeghi is supported by grants from NIH (R01AG065917, R01HL161746) and the Department of Veterans Affairs (I0BX006098). Onur Varli is supported by NIH grant T32HL098069. Jakub Toczek, Mani Salarian, and Mehran Sadeghi are named as inventors on Yale University/Department of Veterans Affairs patent US20240298987A1. S. Michael Yu is a cofounder of 3Helix, which commercializes collagen hybridization peptides. Mehran Sadeghi reports that his spouse is an employee of Boehringer Ingelheim. No other potential conflict of interest relevant to this article was reported.

## Notes

KEY POINTS **QUESTION:** Can denatured collagen serve as an imaging biomarker of collagen remodeling after MI? **PERTINENT FINDINGS:** SPECT/CT using ^99m^Tc-(HE)_3_-(GPO)_9_, a CHP radiotracer, showed higher specific uptake in the infarct zone of mice after MI than in the remote zone and sham controls. Evaluation of collagen production and denaturation after MI revealed a transition from collagen synthesis to remodeling from 3 d to 2 wk after MI. **IMPLICATIONS FOR PATIENT CARE:** ^99m^Tc-(HE)_3_-(GPO)_9_ enables the detection of collagen remodeling after MI, filling a gap in biologic and clinical research that could ultimately improve postinfarction risk stratification and management by monitoring the effect of emerging antifibrotic therapies.

## References

[1] Wynn TA Ramalingam TR. Mechanisms of fibrosis: therapeutic translation for fibrotic disease. Nat Med. 2012;18:1028–1040. 22772564 10.1038/nm.2807 PMC3405917

[2] Ezeani M Noor A Alt K. Collagen-targeted peptides for molecular imaging of diffuse cardiac fibrosis. J Am Heart Assoc. 2021;10:e022139. 34514814 10.1161/JAHA.121.022139 PMC8649514

[3] Frangogiannis NG. Cardiac fibrosis. Cardiovasc Res. 2021;117:1450–1488. 33135058 10.1093/cvr/cvaa324 PMC8152700

[4] Henderson NC Rieder F Wynn TA. Fibrosis: from mechanisms to medicines. Nature. 2020;587:555–566. 33239795 10.1038/s41586-020-2938-9 PMC8034822

[5] Frantz S Hundertmark MJ Schulz-Menger J Bengel FM Bauersachs J. Left ventricular remodelling post-myocardial infarction: pathophysiology, imaging, and novel therapies. Eur Heart J. 2022;43:2549–2561. 35511857 10.1093/eurheartj/ehac223 PMC9336586

[6] Mangion K McComb C Auger DA Epstein FH Berry C. Magnetic resonance imaging of myocardial strain after acute ST-segment-elevation myocardial infarction: a systematic review. Circ Cardiovasc Imaging. 2017;10:e006498. 28733364 10.1161/CIRCIMAGING.117.006498

[7] Arun S Mittal BR Bhattacharya A Rohit MK. Comparison of Tc-99m tetrofosmin myocardial perfusion scintigraphy and exercise F18-FDG imaging in detection of myocardial ischemia in patients with coronary artery disease. J Nucl Cardiol. 2015;22:98–110. 25124826 10.1007/s12350-014-9954-9

[8] Makowski MR Rischpler C Ebersberger U. Multiparametric PET and MRI of myocardial damage after myocardial infarction: correlation of integrin alphavbeta3 expression and myocardial blood flow. Eur J Nucl Med Mol Imaging. 2021;48:1070–1080. 32970218 10.1007/s00259-020-05034-z PMC8041712

[9] Muzard J Sarda-Mantel L Loyau S. Non-invasive molecular imaging of fibrosis using a collagen-targeted peptidomimetic of the platelet collagen receptor glycoprotein VI. PLoS One. 2009;4:e5585. 19440310 10.1371/journal.pone.0005585 PMC2680759

[10] Nahrendorf M Hu K Frantz S. Factor XIII deficiency causes cardiac rupture, impairs wound healing, and aggravates cardiac remodeling in mice with myocardial infarction. Circulation. 2006;113:1196–1202. 16505171 10.1161/CIRCULATIONAHA.105.602094 PMC4066325

[11] Niego B Jupp B Zia NA. Molecular imaging of diffuse cardiac fibrosis with a radiotracer that targets proteolyzed collagen IV. Radiol Cardiothorac Imaging. 2024;6:e230098. 38512024 10.1148/ryct.230098 PMC11056764

[12] Bennink LL Li Y Kim B. Visualizing collagen proteolysis by peptide hybridization: from 3D cell culture to in vivo imaging. Biomaterials. 2018;183:67–76. 30149231 10.1016/j.biomaterials.2018.08.039

[13] Li X Zhang Q Yu SM Li Y. The Chemistry and biology of collagen hybridization. J Am Chem Soc. 2023;145:10901–10916. 37158802 10.1021/jacs.3c00713 PMC10789224

[14] Li Y Foss CA Summerfield DD. Targeting collagen strands by photo-triggered triple-helix hybridization. Proc Natl Acad Sci USA. 2012;109:14767–14772. 22927373 10.1073/pnas.1209721109 PMC3443117

[15] Ahmad AA Ghim M Kukreja G. Collagen hybridizing peptide-based radiotracers for molecular imaging of collagen turnover in pulmonary fibrosis. J Nucl Med. 2025;66:425–433. 39915119 10.2967/jnumed.124.268832 PMC11876730

[16] Jain D Wackers FJ Mattera J McMahon M Sinusas AJ Zaret BL. Biokinetics of technetium-99m-tetrofosmin: myocardial perfusion imaging agent: implications for a one-day imaging protocol. J Nucl Med. 1993;34:1254–1259. 8326381

[17] Ahmad AA Ghim M Toczek J. Multimodality imaging of aortic valve calcification and function in a murine model of calcific aortic valve disease and bicuspid aortic valve. J Nucl Med. 2023;64:1487–1494. 37321825 10.2967/jnumed.123.265516 PMC10478817

[18] Su H Spinale FG Dobrucki LW. Noninvasive targeted imaging of matrix metalloproteinase activation in a murine model of postinfarction remodeling. Circulation. 2005;112:3157–3167. 16275862 10.1161/CIRCULATIONAHA.105.583021

[19] Ashton JR Befera N Clark D. Anatomical and functional imaging of myocardial infarction in mice using micro-CT and eXIA 160 contrast agent. Contrast Media Mol Imaging. 2014;9:161–168. 24523061 10.1002/cmmi.1557 PMC4017375

[20] Toczek J Ye Y Gona K. Preclinical evaluation of RYM1, a matrix metalloproteinase-targeted tracer for imaging aneurysm. J Nucl Med. 2017;58:1318–1323. 28360209 10.2967/jnumed.116.188656 PMC5537616

[21] Travers JG Kamal FA Robbins J Yutzey KE Blaxall BC. Cardiac fibrosis: the fibroblast awakens. Circ Res. 2016;118:1021–1040. 26987915 10.1161/CIRCRESAHA.115.306565 PMC4800485

[22] Beltrami CA Finato N Rocco M. Structural basis of end-stage failure in ischemic cardiomyopathy in humans. Circulation. 1994;89:151–163. 8281642 10.1161/01.cir.89.1.151

[23] Jellis C Martin J Narula J Marwick TH. Assessment of nonischemic myocardial fibrosis. J Am Coll Cardiol. 2010;56:89–97. 20620723 10.1016/j.jacc.2010.02.047

[24] Montesi SB Desogere P Fuchs BC Caravan P. Molecular imaging of fibrosis: recent advances and future directions. J Clin Invest. 2019;129:24–33. 30601139 10.1172/JCI122132 PMC6307954

[25] Stendahl JC Kwan JM Pucar D Sadeghi MM. Radiotracers to address unmet clinical needs in cardiovascular imaging, part 2: inflammation, fibrosis, thrombosis, calcification, and amyloidosis imaging. J Nucl Med. 2022;63:986–994. 35772956 10.2967/jnumed.121.263507 PMC9258561

[26] Barton AK Craig NJ Loganath K. Myocardial fibroblast activation after acute myocardial infarction: a positron emission tomography and magnetic resonance study. J Am Coll Cardiol. 2025;85:578–591. 39772364 10.1016/j.jacc.2024.10.103 PMC11835506

[27] Diekmann J Koenig T Thackeray JT. Cardiac fibroblast activation in patients early after acute myocardial infarction: integration with MR tissue characterization and subsequent functional outcome. J Nucl Med. 2022;63:1415–1423. 35210301 10.2967/jnumed.121.263555 PMC9454470

[28] Varasteh Z Mohanta S Robu S. Molecular imaging of fibroblast activity after myocardial infarction using a ^68^Ga-labeled fibroblast activation protein inhibitor, FAPI-04. J Nucl Med. 2019;60:1743–1749. 31405922 10.2967/jnumed.119.226993 PMC6894377

[29] Zhang J Nie L Razavian M. Molecular imaging of activated matrix metalloproteinases in vascular remodeling. Circulation. 2008;118:1953–1960. 18936327 10.1161/CIRCULATIONAHA.108.789743 PMC2637824

[30] Gona K Toczek J Ye Y. Hydroxamate-based selective macrophage elastase (MMP-12) inhibitors and radiotracers for molecular imaging. J Med Chem. 2020;63:15037–15049. 33206510 10.1021/acs.jmedchem.0c01514 PMC8010999

[31] Mouw JK Ou G Weaver VM. Extracellular matrix assembly: a multiscale deconstruction. Nat Rev Mol Cell Biol. 2014;15:771–785. 25370693 10.1038/nrm3902 PMC4682873

[32] Hwang J Huang Y Burwell TJ. In situ imaging of tissue remodeling with collagen hybridizing peptides. ACS Nano. 2017;11:9825–9835. 28877431 10.1021/acsnano.7b03150 PMC5656977

